# Sustainable Production of Bioethanol Using Levulinic Acid Pretreated Sawdust

**DOI:** 10.3389/fbioe.2022.937838

**Published:** 2022-06-30

**Authors:** Ali Nawaz, Rong Huang, Farah Junaid, Yiwei Feng, Ikram Ul Haq, Hamid Mukhtar, Kankan Jiang

**Affiliations:** ^1^ School of Basic Medical Sciences and Forensic Medicine, Hangzhou Medical College, Hangzhou, China; ^2^ Institute of Industrial Biotechnology, Government College University, Lahore, Pakistan; ^3^ School of Clinical Medicine, Hangzhou Medical College, Hangzhou, China

**Keywords:** deep eutectic solvent, enzymatic saccharification, green biorefinery, levulinic acid, ionic liquids, bioethanol

## Abstract

The sustainability and economic viability of the bioethanol production process from lignocellulosic biomass depend on efficient and effective pretreatment of biomass. Traditional pretreatment strategies implicating the use of mineral acids, alkalis, and organic solvents release toxic effluents and the formation of inhibitory compounds posing detrimental effects on the environment and interfering with the enzymatic saccharification process, respectively. Ionic liquids (ILs) as green solvents were used to overcome this issue, but the deep eutectic solvent as an emerging class of ionic liquids performed better in terms of making the process environmentally and economically viable. The green solvent-based pretreatment strategy applied in the current research was levulinic, acid-based natural deep eutectic solvent (NADES). Three different hydrogen bond acceptors (HBAs)—acetamide, betaine, and choline chloride—in combination with levulinic acid as hydrogen bond donor (HBD) in (HBD: HBA) molar ratio 2:1, were screened for biomass pretreatment. The best deep eutectic solvent was levulinic acid: choline chloride in an optimized molar ratio of 1:0.5, resulting in 91% delignification. The physicochemical parametric optimization of saccharification exhibited maximum enzymatic hydrolysis of 25.87% with 125 mg of pretreated sawdust *via* simultaneous addition of three thermostable cellulases [i.e., endo-1,4-β-D-glucanase (240 U), exo-1,4-β-D-glucanase (180 U), and β-glucosidase (320 U)] for 5 h of incubation at 75°C. The reducing sugar slurry obtained from the saccharified biomass was then added to a fermentation medium for bioethanol production, and a maximum of 11.82% of production was obtained at 30°C, 72 h, and 180 rpm using a 2.5% 24 h old *Saccharomyces cerevisiae* seed culture. The current study revealed that the levulinic-based deep eutectic solvent exhibited remarkable delignification, which led to the efficient enzymatic hydrolysis of sawdust and hence bioethanol production. Furthermore, it will prospect new avenues in bioethanol production using a deep eutectic solvent. Deep eutectic solvent overcame the issues posed by ionic liquids: toxicity, expensive and complex preparation, and non-biodegradability.

## 1 Introduction

Depletion of fossil fuel reserves and increased emission of greenhouse gases leading to climatic changes and global warming have necessitated the notion of alternative bio-based fuel to be practically implemented in the near future. The key step in shifting from a non-renewable to a renewable energy source, which is a shift from petroleum-based to bio-based fuels, is the driving phenomena of biorefineries (Cunha et al., 2020). Biorefinery is defined as the equivalent renewable of a petroleum refinery, the difference being in the starting raw material. Biorefinery converts the raw material into a wide variety of chemicals and energy carriers, which can lead to the development of the circular economy. The biorefinery concept is based on lignocellulosic materials, which produce bio-based products that are recoverable (to a certain degree) and recyclable. Biorefineries have made a fine amalgam of green chemistry, keeping in view the environmental impact of fuel. This amalgam aims to limit or minimize the use and generation of hazardous chemicals (Capolupo and Faraco, 2016). Therefore, sustainable bioethanol production can be achieved using green solvents that are deep eutectic solvents, so the end product of green biorefineries is environmentally benign and recyclable and produces minimum waste.

Lignocellulosic biomass is an inexhaustible biomaterial, mainly composed of lignin, hemicellulose, cellulose, and extractives in different proportions (Huang et al., 2022a). Sawdust from the sawmill industry is a potential, cost-efficient raw material to produce bioethanol. The use of sawdust for biofuel production promotes the local valorization of wood waste, establishing the concept of forest biorefinery (Alio et al., 2021). From the bioethanol production perspective, lignin is a barrier to enzymatic hydrolysis because it irreversibly binds to the cellulases rendering enzyme adsorption on cellulose (Takada et al., 2020; Huang et al., 2022b). Pretreatment is employed to disintegrate the cross-linked biomass fractions, enhancing the biodegradability and ease of access of hemicellulose and cellulose for enzymatic hydrolysis (Robak and Balcerek, 2020).

The choice of an appropriate pretreatment method is critical in terms of determining the sustenance and the economic viability of a project. It is evident from the literature that the previous pretreatment techniques were more concentrated on the techno-economic viability than on the sustenance of the environment (Vieira et al., 2020). The green solvent used in this research is deep eutectic solvent (DES), acknowledged as a class of ionic liquids (ILs). Deep eutectic solvents are defined as a group of large, non-symmetrical ions with low lattice energy and hence low melting points (Smith et al., 2014). They are prepared *via* complexation or a combination of hydrogen bond donors (HBDs) (alcohols, amides, amines, or carboxylic acids) and quaternary ammonium salts as hydrogen bond acceptors (HBA) at a moderate temperature of 60°C –80°C in a certain ratio to form a eutectic mixture (Satlewal et al., 2018).

Deep eutectic solvents and ionic liquids are alike in their physical properties but have varying chemical properties. Both exhibits low or negligible vapor pressure, non-flammability, and a wide liquid range. Ionic liquids have wide electrochemical windows and high dissolution ability, used in biomass dissolution and conversion as a solvent and a catalyst (Smith et al., 2014; Chen and Mu, 2019). The drawbacks that hold back ILs as a green solvent are their complex, expensive preparation, toxicity, and non-biodegradability. On the contrary, these deep eutectic solvents are cost-effective, easily prepared, potentially biodegradable, innocuous, and safe (Zhang et al., 2020). Deep eutectic solvents fulfill the twelve principles of green chemistry, which entails their use in the sustainable pretreatment of biomass. They have high air stability, thermal stability, low volatility, non-inflammability, and high purity (Xu et al., 2020).

The molar ratio of HBD and HBA is closely related in the context of their potential to remove lignin and hemicellulose or their dissolution ability of chemical components, that is, lignin and cellulose, subsequently influencing the saccharification process/enzymatic hydrolysis of the pretreated substrate (Xu et al., 2020). Saccharification of pretreated lignocellulosic biomass is another crucial step in the bioconversion of substrate into the desired end product, ethanol, *via* releasing fermentable sugars from crystalline cellulose and hemicellulose (Kucharska et al., 2020). Enzymatic saccharification/hydrolysis is usually carried out *via* cellulases and hemicellulases. Cellulases are commonly used to refer to the three enzymes that convert cellulose into glucose (fermentable monosaccharide): endocellulase, exocellulase, and glucosidase (Zhao et al., 2019). Different physicochemical parameters affect hydrolysis efficiency, such as incubation temperature, pH, agitation speed/rpm, incubation time, enzyme/substrate ratio, and particle size (Chavan and Gaikwad, 2021; Faizal et al., 2021).

Separate hydrolysis and fermentation (SHF) is the most studied technique in the bioethanol production process. This process enables independent optimization of the saccharification step for maximal sugar release and the fermentation step for ethanol production (Moodley and Gueguim Kana, 2019). Limitations of this strategy are inhibition of cellulases by cellobiose and glucose and the potential risk of contamination due to the prolonged duration of saccharification (Keshav et al., 2021).

Maugeri and Domı (2012) first prepared choline chloride: levulinic acid DES in equimolar concentration, but exploitation of DES for biomass pretreatment was first reported by Gunny et al. (2014). Several reports were available with choline chloride-based DES pretreatment of lignocellulosic biomass (Wang et al., 2020). However, reports regarding levulinic acid-based DES are infrequent (Ling et al., 2020). By considering the research gap, the current research work focuses on selecting HBA for levulinic acid-based DES pretreatment, optimization of the molar ratio (HBD:HBA), and physicochemical parametric optimization of saccharification and bioethanol production.

## 2 Materials and Methods

### 2.1 Thermophilic Cellulases

The thermophilic cellulases, endo-1,4*-* β -glucanase (E.C. 3.2.1.4) of *Thermotoga naphthophila*, and exo-1,4- β -glucanase (E.C. 3.2.1.91) and β -1,4-glucosidase (E.C. 3.2.1.21) of *Thermotoga petrophila* cloned in *Escherichia coli* were obtained from the project entitled “Production of Bioenergy From Plant Biomass” at the Institute of Industrial Biotechnology, GC University Lahore, Pakistan.

### 2.2 Substrate

Sawdust was acquired from the local furniture market of Lahore, Punjab, Pakistan. The biomass was washed, dried, and sieved with mesh size 400 to attain homogeneously sized particles.

### 2.3 DES Preparation and Pretreatment

Levulinic acid-based DES with three variable HBAs (i.e., acetamide, betaine, and choline chloride) was prepared at an initial molar ratio of 2:1 (HBD: HBA) in screw-capped reagent bottles and kept in a shaking water bath, 80°C, 120 rpm for 1–2 h till complete dissolution and appearance of clear solution (Ling et al., 2020). After selecting the appropriate HBA for DES, molar ratios of both HBA and HBD varied from 0.1 to 2.5 M.

Pretreatment was conducted for 1 g/100 ml of DES in a screw-capped reagent bottle at 121°C, 15 psi for 30 min. Upon pretreatment, the pretreated sample was separated from DES by filtration. The filtered pretreated biomass was washed to neutral pH to remove all the residual DES.

### 2.4 Lignocellulosic Content Estimation

The lignin content of untreated and pretreated biomass was estimated using the following equation (Irfan et al., 2011):

$$Lignin\;\left(\%\right)=\frac{Lignin\;weight\;\left(g\right)}{Biomass\;\left(g\right)}\;\times 100.$$

The percentage delignification was calculated using the following formula:

$$\mathrm{Delignification}\;\left(\%\right)=\;\frac{L1-\;L2}{L1}\times 100,$$

L1 = lignin content of control (untreated substrate).

L2 = lignin content of the pretreated substrate.

Cellulosic content on a dry matter basis was estimated using the method of Gopal and Ranjhan (1980) using the following equation:

$$Cellulose\left(DM\;basis\right)=\frac{Weight\;of\;digested\;material-Weight\;of\;ash}{Weight\;of\;substrate\;\left(DM\;basis\right)}\times 100.$$

The hemicellulosic content of the pretreated and untreated substrate was determined by NDF and ADF treatment (Van Soest and Roberston, 1979):

% Hemicellulose =NDF−ADF.

### 2.5 Enzymatic Saccharification

The saccharification of pretreated sawdust was conducted by taking 100 mg of the pretreated substrate in a screw-capped reagent bottle. Cellulases, endo-1,4- β -glucanase (90 U), exo-1,4- β -glucanase (80 U), and β -1,4-glucosidase (220 U) were taken in experimental and control (lacking substrate) reagent bottles, incubated at 85 in shaking water 50 rpm. After 2 h of regular intervals, each cellulase was added. The samples were withdrawn at a regular interval of 1 h to estimate the release of reducing sugar *via* the DNS method of Miller (1959). The percentage saccharification was determined using the proposed equation of Vallander and Erikson (1987):

$$\%\;Saccharification=\frac{R.S\times V\times F1}{M\times F2}\times 100\;.$$

### 2.6 Effect of Physicochemical Parameters on Saccharification

The physicochemical parameters optimized for saccharification were sequential/simultaneous addition of cellulases, incubation time (1–7 h), incubation temperature (70 °C –90°C), substrate concentration (50–200 mg), concentration of endo-1,4- β -glucanase (40–290 U), exo-1,4- β -glucanase (30–230 U), and β -glucosidase (120–370 U).

### 2.7 Preparation of Seed Inoculum for Ethanol Fermentation

The vegetative seed inoculum of *Saccharomyces cerevisiae* (IIB-56) acquired from the culture bank of the Institute of Industrial Biotechnology, GC University Lahore, Pakistan, was prepared by aseptically transferring dry (granulated) baker’s yeast into a 50 ml medium containing 10 g glucose, 0.25 g yeast extract, and 0.15 g ammonium sulfate. The flask was incubated at 30 C, 180 rpm for 24 h.

### 2.8 Submerged Fermentation for Ethanol Production

The submerged fermentation for ethanol production was carried out in a fermentation medium using the reducing sugar slurry (in place of glucose) obtained after optimization. The fermentation medium was inoculated with 2.5% of 24 h seed inoculum of *S. cerevisiae* aseptically, incubated at 30°C, 180 rpm for 72 h. The samples were harvested at regular intervals to estimate the ethanol content during fermentation (Yuan et al., 2011).

### 2.9 Statistical Analysis

The experiment was run in triplicate. Statistical analysis was done using SPSS version 16.00 (IBM Analytics, New York, United States). One-way ANOVA was applied on replicates to observe the significant difference with the probability (*p*) value. Error bars in the figures of the Results section indicated standard deviation ( ±SD) among the replicates run, varying significantly at *p* < 0.05.

## 3 Results

### 3.1 Pretreatment of Lignocellulosic Biomass (Sawdust)

**FIGURE 1:**
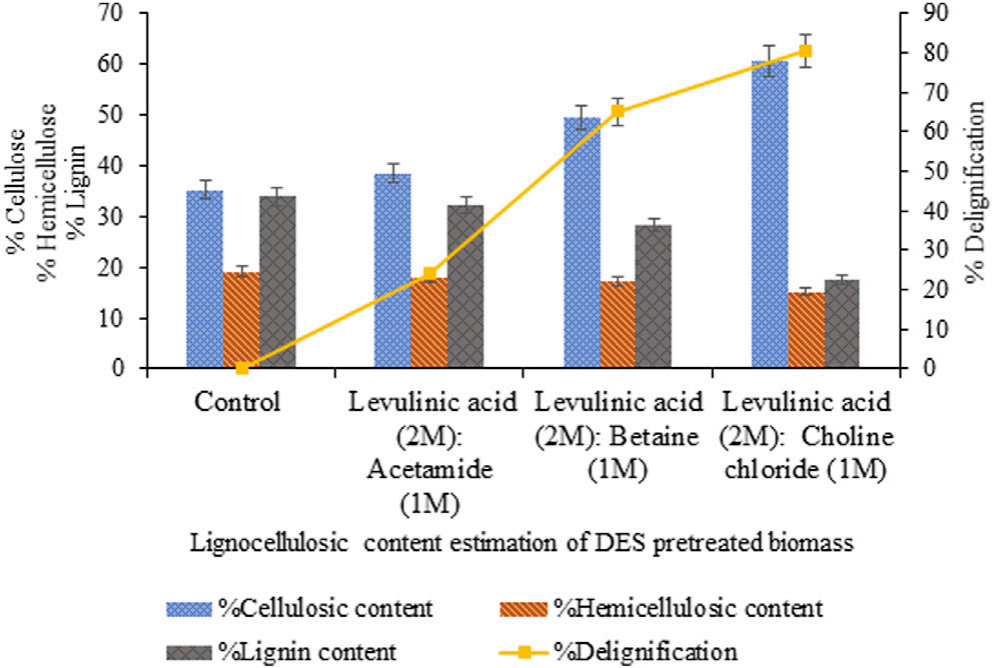
Content estimation of sawdust pretreated with levulinic acid-based DESs with varying HBAs.

The pretreatment of sawdust with levulinic acid-based DES prepared with three variable HBAs exhibited maximum delignification (80.5%, *p* < 0.05) and cellulosic content (60.7%, *p* < 0.05) for levulinic acid: choline chloride DES (2:1) compared to levulinic acid: betaine, levulinic acid: acetamide DES exhibiting 65% (*p* < 0.05) and 24% (*p* < 0.05) delignification, as shown in Figure 1.

**FIGURE 2:**
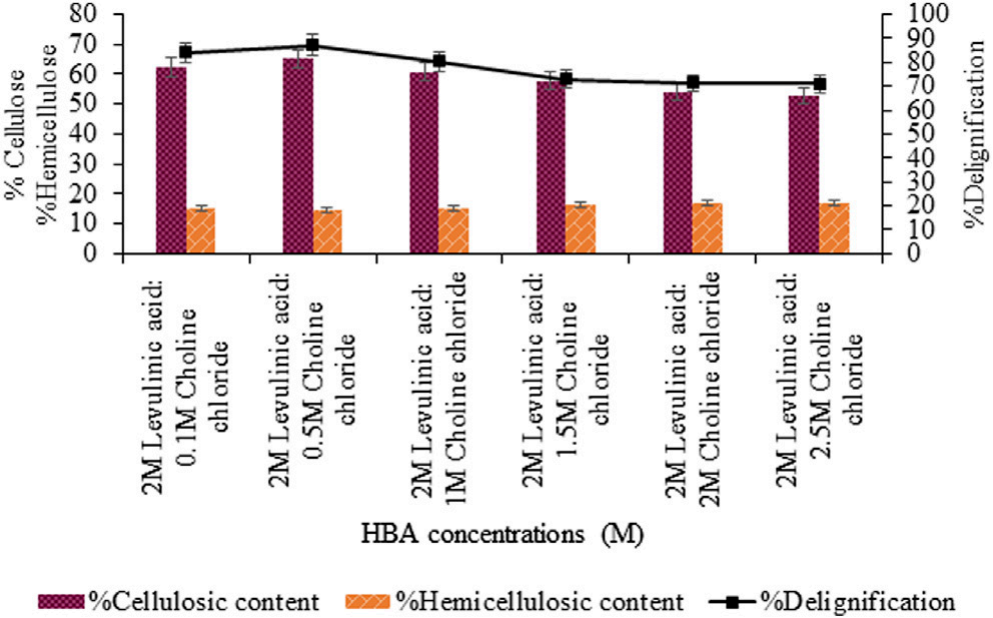
Content estimation of levulinic acid-based pretreatment with varying choline chloride concentrations (M).

**FIGURE 3:**
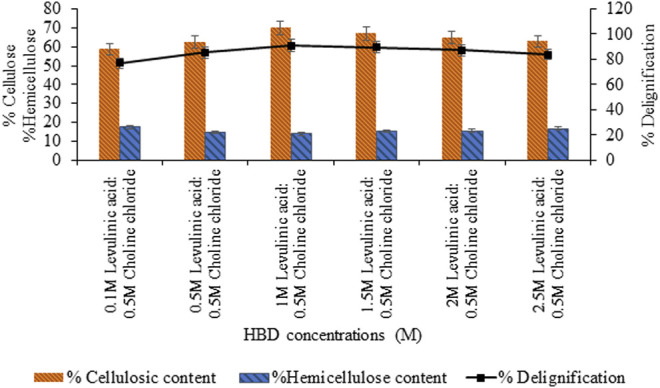
Content estimation of sawdust pretreated with varying concentrations of levulinic acid.

Further optimization of the molar ratio of HBA, that is, choline chloride varying from 0.1 to 2.5 M, showed maximum delignification (87.1%, *p* < 0.05) for 0.5 M choline chloride: 2 M levulinic acid based-DES shown in Figure 2. However, upon varying concentration of levulinic acid (0.1–2.5 M), maximum delignification (90.1%, *p* < 0.05) and cellulosic content (70.16%, *p* < 0.05) was recorded for the 1:0.5 M concentration of levulinic acid and choline chloride DES (Figure 3).

### 3.2 Enzymatic Saccharification of Pretreated Sawdust

**FIGURE 4:**
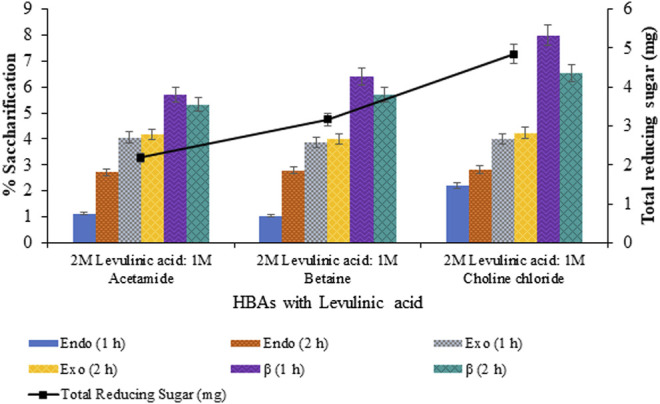
Saccharification of sawdust pretreated with varying HBAs in DES.

**FIGURE 5:**
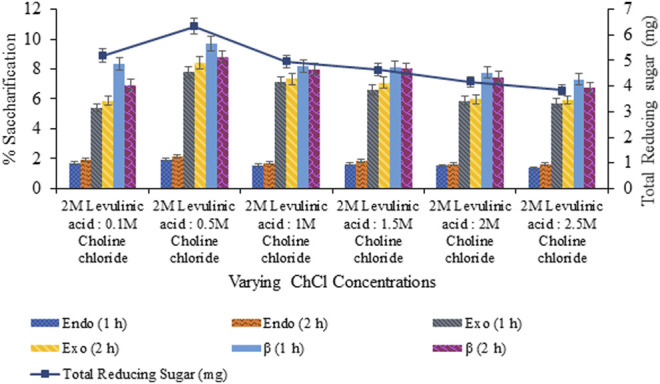
Saccharification of sawdust pretreated with varying choline chloride concentrations (0.1–2.5 M).

**FIGURE 6:**
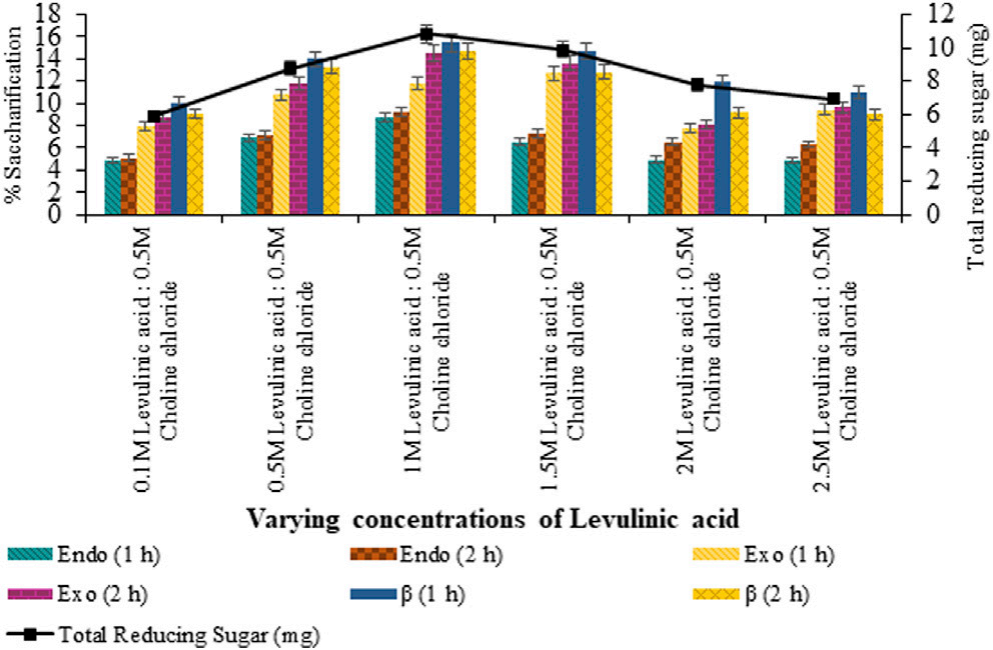
Saccharification of sawdust pretreated with varying levulinic acid concentrations (0.1–2.5 M).

Saccharification of pretreated sawdust with variable HBAs and molar ratios of DES constituents resulted in maximum saccharification (7.99%; *p* < 0.05) for levulinic acid: choline chloride (2:1) pretreated sawdust (Figure 4). However, upon the varying concentration of HBA of selected DES, maximum saccharification of 9.73% (*p* < 0.05) was recorded for levulinic acid: choline chloride (2:0.5) and 15.44% (*p* < 0.05) for levulinic acid: choline chloride (1:0.5), upon the varying concentration of HBD as evident from Figures 5, 6 respectively.

### 3.3 Effects of Physicochemical Parameters on Enzymatic Saccharification

#### 3.3.1 Effect of Cellulase Addition on Saccharification

**FIGURE 7:**
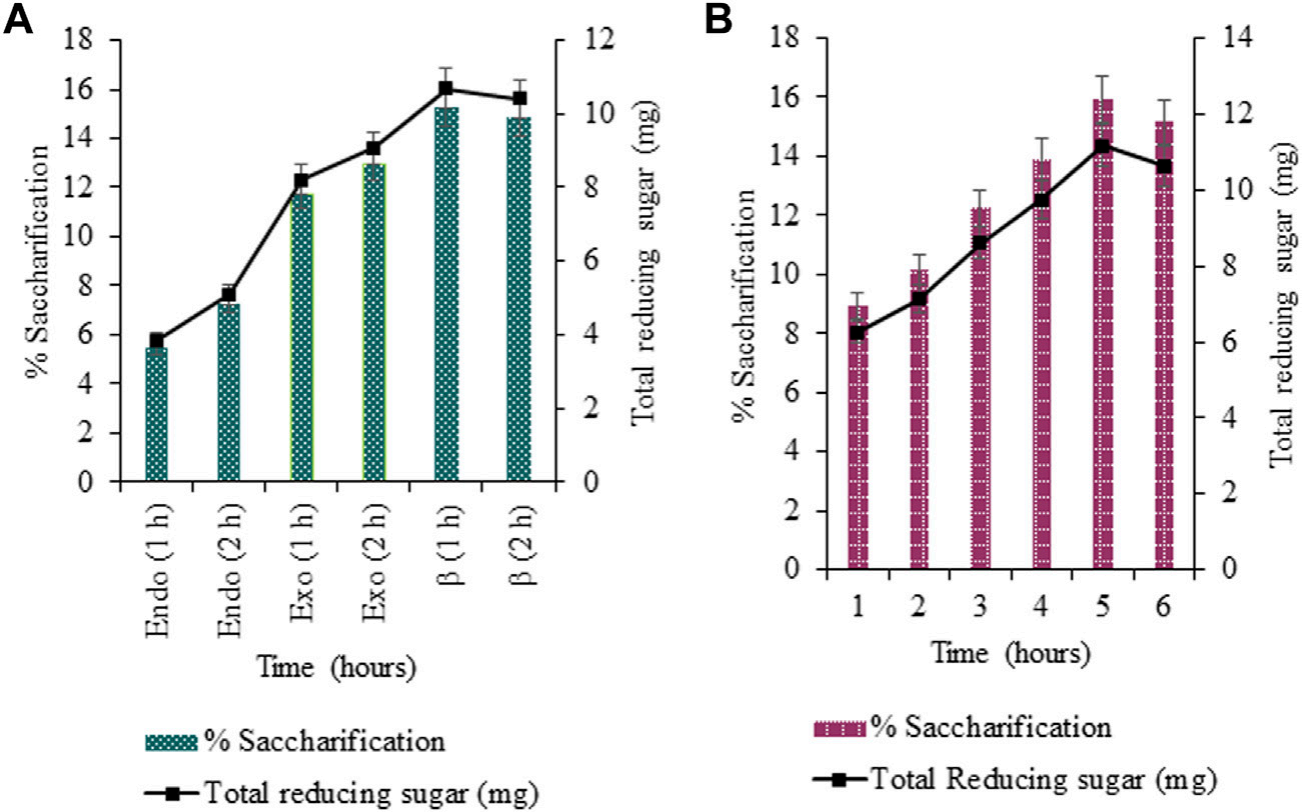
Effect on simultaneous **(A)** and sequential **(B)** addition of cellulases on saccharification.

The effect of cellulase addition was evaluated by adding cellulases simultaneously (all at once) and sequentially (after a regular interval of 2 h) for 6 h; at 85°C; 50 rpm. Maximum saccharification for simultaneous and sequential addition of cellulases was 15.93% (*p* < 0.05) and 15.23% (*p* < 0.05) respectively, as exhibited in Figure 7 (Figures 7A,B). Thus, the simultaneous addition of cellulases exhibited better hydrolysis than the sequential one.

#### 3.3.2 Optimization of Incubation Time for Saccharification

**FIGURE 8:**
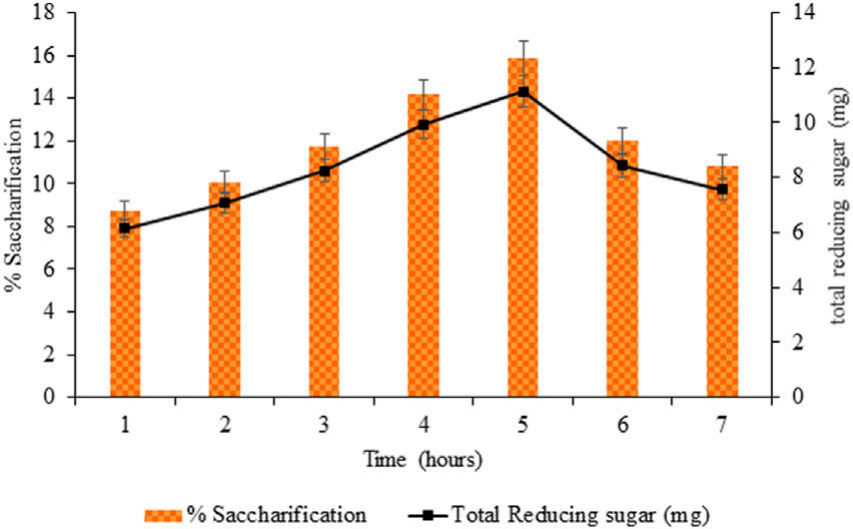
Optimization of incubation time for saccharification.

The incubation time of the simultaneously added cellulase mixture for saccharification was 5 h, exhibiting maximum saccharification and total reducing sugar of 15.88% (*p* < 0.05) and 11.44 mg (*p* < 0.05), respectively (Figure 8).

#### 3.3.3 Effect of Incubation Temperature on Saccharification

**FIGURE 9:**
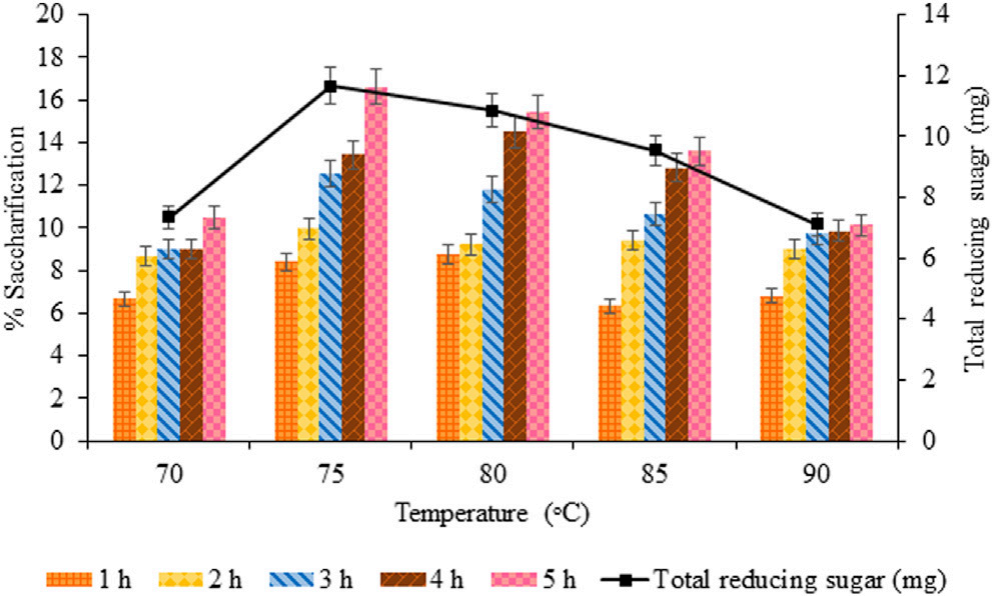
Effect of incubation temperature on saccharification.

For thermophilic cellulases, temperature regimes (70°C–90°C) with a regular increment of 5°C were used to evaluate the optimum saccharification of biomass. Maximum saccharification (16.62%, *p* < 0.05) and total reducing sugar (11.66 mg, *p* < 0.05) was estimated at 75°C, 5 h, 50 rpm, using 100 mg of pretreated sawdust, evident from Figure 9.

#### 3.3.4 Optimization of Substrate Concentration for Saccharification

**FIGURE 10:**
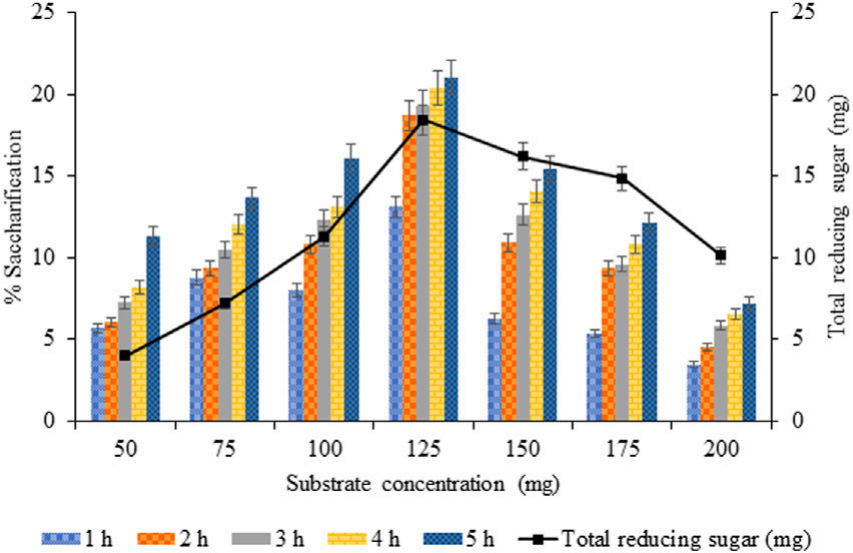
Optimization of substrate concentration for saccharification.

By increasing substrate concentration from 50 to 125 mg, saccharification also increased from 11.31% (*p* < 0.05) to 20.99% (*p* < 0.05). However, a further increase in biomass concentration decreased saccharification to 7.22%, but the total reducing sugar did not follow the same trend (Figure 10). Thus, substrate concentration of 125 mg was optimum with maximum saccharification (20.99%, *p* < 0.05) and release of total reducing sugar (18.41 mg, *p* < 0.05).

#### 3.3.5 Effect of Endo-1,4- β -Glucanase on Enzymatic Saccharification

**FIGURE 11:**
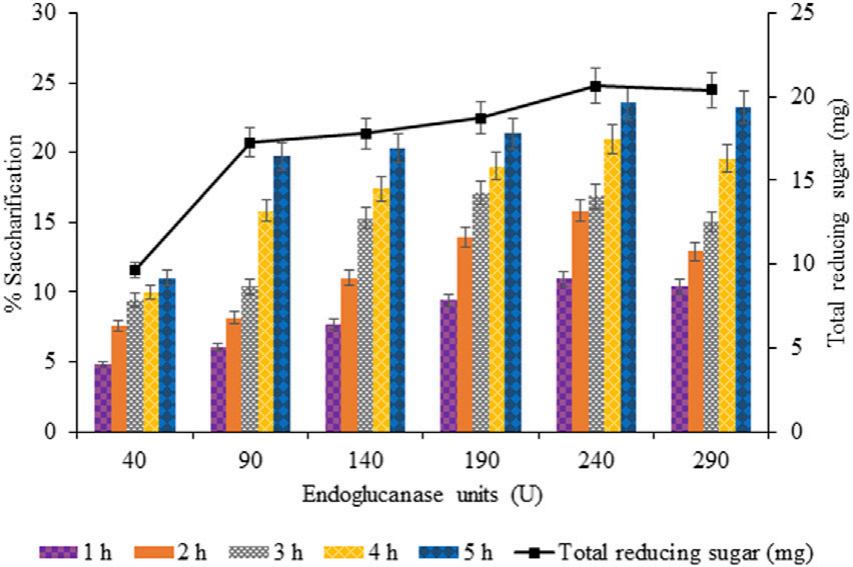
Effect of endo-1,4- β -glucanase (40–290 U) on enzymatic saccharification.

The units of endoglucanase varied from 40 to 290 U with a regular increment of 50 U to analyze the concentration required for optimal saccharification while keeping the units of the remaining two cellulases constant. Endoglucanase (240 U) gave maximum saccharification of 23.55% (*p* < 0.05), upon 5 h of incubation at 75°C using 125 mg of pretreated sawdust (Figure 11).

#### 3.3.6 Effect of Exo-1,4- β -Glucanase on Saccharification

**FIGURE 12:**
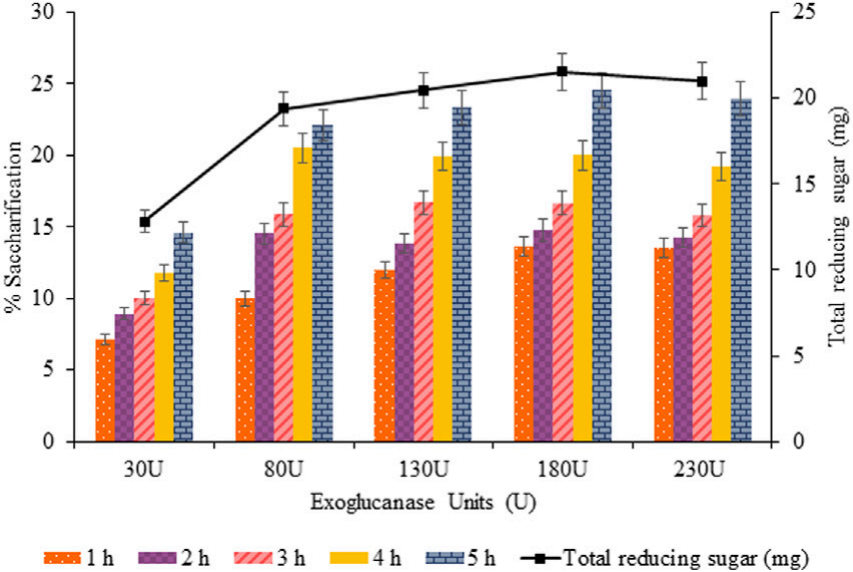
Effect of exo-1,4- β -glucanase (30–230 U) on saccharification.

The units of exoglucanase varied from 30 to 230 U with a regular increment of 50 U to evaluate the optimum enzyme units for enzymatic hydrolysis. The maximum saccharification and total reducing sugar was 24.54% (*p* < 0.05) and 21.52 mg (*p* < 0.05) for 180 U of exoglucanase with 240 U of endoglucanase and 220 U of β -glucosidase, as shown in Figure 12.

#### 3.3.7 Effect of β -Glucosidase on Enzymatic Hydrolysis

**FIGURE 13:**
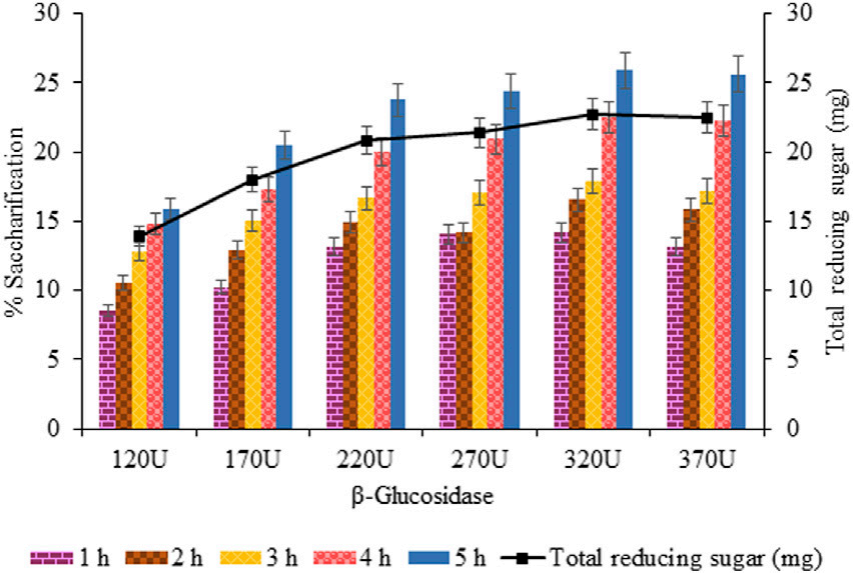
Effect of β -glucosidase (120–370 U) on enzymatic hydrolysis.

β -Glucosidase is responsible for producing fermentable sugar (i.e., glucose) to be used to produce ethanol. β -Glucosidase (320 U) was estimated for maximum saccharification (25.87%, *p* < 0.05) and the highest release of total reducing sugar (22.69 mg, *p* < 0.05) with optimized units of endoglucanase (240 U) and exoglucanase (180 U). Upon increasing the concentration, saccharification dropped (25.62%, *p* < 0.05), evident from Figure 13.

### 3.4 Fermentation of Saccharides Into Ethanol

**FIGURE 14:**
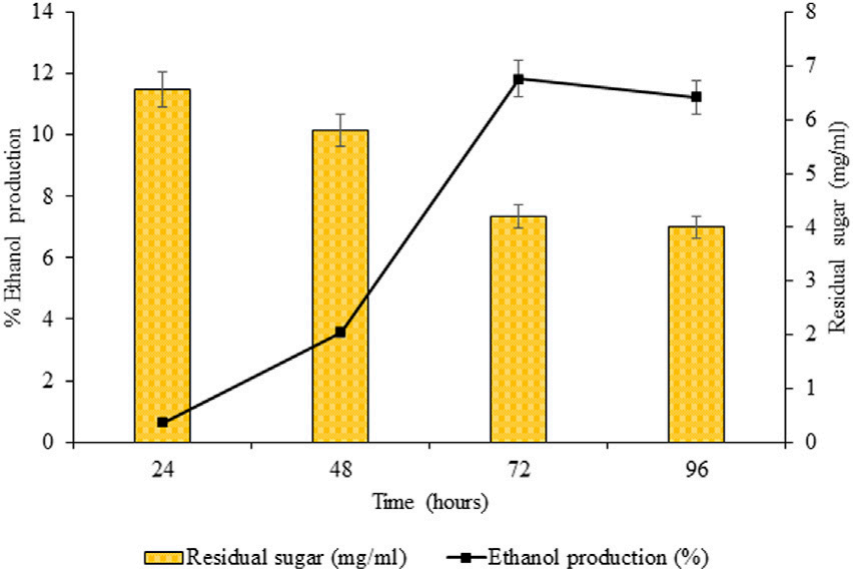
Fermentation of saccharides into ethanol.

The released reducing sugars obtained after optimized saccharification of pretreated sawdust were subjected to the final step of ethanol production by *S. cerevisiae*. The ethanol production was estimated by potassium dichromate reagent, which turned green from orange, indicating the presence of ethanol in the supernatant. Maximum ethanol production (11.82%, *p* < 0.05) was obtained after 72 h of incubation at 30°C, 180 rpm. Initially, ethanol production estimated after 24 h was not appreciable (0.647%, *p* < 0.05), but after 48 h, a noticeable increase in ethanol production was estimated. Upon 96 h of incubation, ethanol production (11.23%, *p* < 0.05) was observed to decline (Figure 14).

## 4 Discussion

The sustainability and the economic viability of bioethanol production were achieved *via* lignocellulosic biomass (sawdust) and novel levulinic acid-based pretreatment applied in current research. Pretreatment is the key bottleneck and the costliest step in the sustenance of such projects. DES pretreatment resolved the issue. The DES used in the current research were natural deep eutectic solvents belonging to Class III, reported to be used in biomass valorization (Scelsi et al., 2021). Levulinic acid as HBD was used because it had a single carboxyl group; the second functional group was a ketonic group that interacted with lignin rather than the carboxyl group, which was responsible for the reduction in lignin solubility (Soares et al., 2017; Magalhães et al., 2021). Three hydrogen bond acceptors—acetamide, betaine, and choline chloride—were of natural origin to prepare natural deep eutectic solvent (NADES) to out rule the potential environmental hazards posed by ILs and other organic solvents used for pretreatment (Scelsi et al., 2021).

The efficient removal of lignin and hemicellulose depends on the cleavage of a covalent bond between lignin and hemicellulose containing phenyl glucoside, benzyl ester/ether groups, and cross-linking hydrogen bonds. The strong hydrogen bond in lignocellulosic biomass might be weakened by a competing hydrogen bond formed between the chloride ions from choline chloride constituting DES and the hydroxyl group of lignin and carbohydrate. Thus, breaking the lignin carbohydrate complexes and removing lignin and hemicellulose in DES during pretreatment (Liu et al., 2017; Smink et al., 2019). On this basis, levulinic acid: choline chloride DES exhibited better potential to remove lignin and hemicellulose than levulinic acid: betaine and levulinic acid: acetamide DESs. Similarly, in the literature, a few articles reported the exploitation of levulinic acid and keto-organic acid in DES preparation used for biomass valorization.

The molar ratio of HBD (i.e., levulinic acid) and selected hydrogen bond acceptor (HBA) (i.e., choline chloride) was optimized to be 1:0.5 whereas, the reported molar ratio for this combination was 2:1 by Ling et al. (2020) and 1:1 by Alvarez-Vasco et al. (2016). The molar ratio of levulinic acid (HBD) to choline chloride (HBA) was responsible for affecting the strength of the hydrogen bond in the resulting DES. The strength of the DES hydrogen bond, as a result, affects their mobility as well as the degradation potential of biomass in pretreatment (Xu et al., 2021). Therefore, maybe the molar ratio of 1:0.5 exhibited strong hydrogen bonding compared to the other variable ratios of HBD to HBA. Thus, pretreatment intensity was best at this molar ratio of HBD to HBA forming DES, that is, 91% of delignification and cellulosic content of 70.16%.

After the pretreatment process, enzymatic hydrolysis/saccharification of lignocellulosic biomass was recognized as the techno-economic bottleneck in converting lignocellulosic biomass into ethanol (Alio et al., 2020). The type of pretreatment given to the substrate was among the factors influencing saccharification (Vasić et al., 2021). This was evident from the result of saccharification for DES pretreatment with variable HBAs and varying concentrations of HBA and HBD. Chourasia et al. (2021) reported efficient saccharification of choline chloride-based DES pretreated biomass. Ling et al. (2020) also reported maximum enzymatic hydrolysis for the pretreated biomass exhibiting maximum delignification with levulinic acid: choline chloride DES. The maximum saccharification of 15.44% for the optimized levulinic acid and choline chloride molar ratio of 1:0.5 from the initial pretreatment molar ratio of 2:1 was 7.99%. Thus, the adsorption of cellulases to exposed cellulose after efficient delignification increased saccharification. The decrease in percentage saccharification might be due to the deposition of lignin and pseudo lignin droplets on the surface of pretreated biomass, due to which enzymes adsorbs unproductively on the substrate (Lin et al., 2021).

The addition of cellulases played a critical role in saccharification; three basic cellulases (endoglucanases, exoglucanases, and β -glucosidases) were employed to deconstruct cellulose into reducing sugars. In the current research, the simultaneous addition of cellulases (i.e., a blend of three cellulases) gave higher saccharification compared to the sequential addition of cellulases for 90 U of endoglucanase, 80 U of exoglucanase, and 220 U of β -glucosidase. Obeng et al. (2018) and Malgas et al. (2020) also reported cellulase synergism for a cocktail of cellulases in enzymatic hydrolysis of biomass. However, the blend ratio and enzyme source were different, which might be responsible for the variation in results. However, a commercial preparation of cellulases also supports the simultaneous addition of cellulases for their synergistic action in saccharification compared to their sequential addition.

Incubation time for enzymatic hydrolysis determines the extent of contact between enzyme (i.e., cellulase) and pretreated substrate (sawdust) molecules involved in the reaction, affecting the rate of product formation (i.e., reducing sugar). After 5 h of incubation, saccharification decreased due to the exhaustion of amorphous cellulose by cellulases attack in the initial stages and the deferred hydrolysis rate of crystalline cellulose (Sridevi et al., 2015).

In the current research, the thermostable cellulase sourced from *Thermotoga petrophila* cloned in *Escherichia coli* exhibited maximum enzymatic hydrolysis of 16.62% at 75°C. According to Arrhenius’s theory, the rate of enzyme-catalyzed reaction rises with increasing temperature but up to a certain limit. The kinetic energy of reacting molecules increases with the temperature rise, resulting in a higher collision rate and subsequent substrate conversion into a product. However, at elevated temperature, water that critically affects protein folding and structure are lost, due to which enzyme activity is compromised (Chavan and Gaikwad, 2021). The deviations were possibly due to the difference in the substrate used in enzyme assay and saccharification, as cellulases exhibit differential specificity and affinity for soluble and insoluble substrates (Sidar et al., 2020). Most of the thermophilic microbes did not have the potential to degrade crystalline cellulose due to the lack of cellulose-binding modules (CBMs) (Maki et al., 2009). The half-life of cellulases has been reported to be 8 h at 80°C (with optimum pH) by Wang et al. (2011), so upon increasing temperature, the half-life decreases. That is why the saccharification dropped at elevated temperatures.

The maximum substrate concentration of 125 mg exhibited the highest saccharification yield (20.99%), and a further increase in the substrate was not supportive in increasing saccharification percentage and release of reducing sugar. A similar effect of increasing substrate concentration resulting in deferred conversion rate/enzymatic hydrolysis was justifiable for the factors limiting the substrate conversion into the product as inadequate stirring for higher substrate loadings, increased viscosity, deferred seepage of cellulases to cellulose, and mass transfer limitations (Althuri and Banerjee, 2019; Ailo et al., 2020).

The saccharification process of pretreated sawdust was influenced by substrate-related factors and other physical conditions, and enzyme-related factors were also responsible for affecting the hydrolysis rate positively and negatively. In the current findings, the optimized concentration of crude cellulases (i.e., endoglucanase, exoglucanase, and β -glucosidase) was 240, 180, and 320 U for increasing the saccharification percentage from 20.99 to 25.87 (4.88%). Upon further increasing the concentration of each cellulase, no further increase was observed, although a slight decrease in percentage saccharification was recorded.

The long-chain oligosaccharides (cello-oligosaccharides) are the end product of endoglucanases, which are then processed by exoglucanases, and the resulting disaccharides (cellodextrins/cellobiose) are then cleaved by β -glucosidases. Thus, endoglucanase and exoglucanase activity is the rate-limiting step in this substrate processivity and channeling *via* cellulases interactions. Therefore, the optimal amount of exoglucanase was required for a higher endoglucanase titer to prevent feedback inhibition. Similarly, a higher titer of glucosidase was required to prevent the feedback inhibition of endoglucanases and exoglucanases by cellobiose accumulation (Obeng et al., 2018).

The jamming effect was created by the overcrowding of cellobiohydrolases (CBH) in the crystalline cellulose due to the orientation of cellulose fiber and restricted movement of enzymes over it in one direction (i.e., along with the fiber). That is why upon increasing the concentration of cellulases, several molecules bound adjacent to each other over the exposed cellulosic content tend to impede each other. As a result, not all enzymes were able to move at the same pace, causing a reduction in cellulose to glucose conversion rate (Bommarius et al., 2008).

However, further increase in enzyme concentration was not supportive in increasing percentage saccharification probably due to the following reasons: source of cellulases, product inhibition, synergism among cellulases, non-specific binding, and specific activity of enzymes, as well as their processivity and compatibility with the substrate to be saccharified (Yang et al., 2011). However, other reasons were the increased rate of transglycosylation reactions, inadequate mixing, hydrodynamic instability, and slurry suspension (Alrumman, 2016).

The bioethanol production of 11.82% using *S. cerevisiae,* with a maximum consumption of reducing sugar slurry, was obtained within 72 h. After 72 h, a slight decrease in glucose consumption and its subsequent conversion into ethanol was observed. The decrease might be due to the depletion of essential nutrients supporting yeast growth and the accumulation of toxic metabolites. Alio et al. (2020) reported ethanol production of 16 g/L from saccharified biomass with substrate (sawmill mixed feedstock) loading of 7.5% using *S. cerevisiae.* However, with DES pretreated sorghum straw, Wu et al. (2021) reported 0.45 g ethanol/g glucose using *S. cerevisiae*. Thus, variation in results was due to the difference in the substrate, using saccharified biomass slurry of reducing sugar instead of saccharified biomass.

## Data Availability

The raw data supporting the conclusion of this article will be made available by the authors without undue reservation.
